# Alexithymia and peer victimisation: interconnected pathways to adolescent non-suicidal self-injury

**DOI:** 10.1192/bjo.2023.653

**Published:** 2024-02-12

**Authors:** Qian-Nan Ruan, Linhui Liu, Guang-Hui Shen, Yu-Wei Wu, Wen-Jing Yan

**Affiliations:** Wenzhou Seventh People's Hospital, Wenzhou, China; Lishui Second People's Hospital, Affiliated to Wenzhou Medical University, Lishui, China; Student Affairs Division, Wenzhou Business College, Wenzhou, China; Wenzhou Medical University, Wenzhou, China; and Zhejiang Provincial Clinical Research Centre for Mental Illness, Affiliated Kangning Hospital, Wenzhou Medical University, Wenzhou, China

**Keywords:** Alexithymia, non-suicidal self-injury (NSSI), peer victimisation, adolescents, emotional dysregulation

## Abstract

**Background:**

The prevalence of non-suicidal self-injury (NSSI) among adolescents underscores the importance of understanding the complex factors that drive this behaviour. Framed within broader constructs of emotional regulation theories, alexithymia and peer victimisation are thought to interact to influence NSSI behaviours.

**Aim:**

This research addresses whether alexithymia and peer victimisation serve as risk factors for NSSI and, if so, how these factors interact with each other.

**Method:**

This quantitative study analysed data from 605 adolescents, using a range of validated self-report measures including the Toronto Alexithymia Scale. Statistical analyses including one-way analysis of variance, multiple regression and structural equation modelling were employed to scrutinise the relationships among the variables.

**Results:**

Alexithymia and peer victimisation significantly predicted NSSI behaviours. Specifically, the ‘difficulty in identifying feelings’ subscale of alexithymia emerged as a noteworthy predictor of NSSI (*P* < 0.001). Peer victimisation mediated the relationship between alexithymia and NSSI, explaining approximately 24.50% of alexithymia's total effect on NSSI. In addition, age was a significant predictor of NSSI, but gender and education years were not (*P* > 0.05). These relationships were found to be invariant across genders.

**Conclusions:**

This study enriches our understanding of the interplay between alexithymia, peer victimisation and NSSI, particularly within the Chinese context. Its findings have significant implications for a rethinking of alexithymia's theoretical construct and interventions targeting emotional literacy and peer dynamics among adolescents. Future research could benefit from a longitudinal design to establish causality.

Non-suicidal self-injury (NSSI), defined as the deliberate act of harming oneself without evident suicidal intent, has received increasing attention in recent years owing to its rising prevalence, especially among adolescents.^1^ According to a comprehensive review,^2^ approximately 17% of adolescents globally have engaged in NSSI at least once in their lifetimes, highlighting its significance as a public health concern. This prevalence underscores the importance of understanding the complex factors that drive this behaviour. The study of NSSI in adolescents has resulted in a considerable body of research, elucidating various risk factors and determinants.^3^ Among these, alexithymia and peer victimisation have been recurrently flagged as crucial variables.^4,5^

Alexithymia, a term derived from the Greek words meaning ‘no words for emotions,’ refers to a multifaceted personality construct characterised by difficulties in identifying and describing one's feelings, a constricted imaginal capacity and an externally oriented cognitive style.^6,7^ Individuals with high alexithymic traits often struggle with understanding, processing and communicating their emotions, making it challenging for them to navigate emotional situations effectively.^8^ Notably, research has increasingly highlighted the link between alexithymia and various maladaptive behaviours, including NSSI. For instance, a study^9^ found that individuals exhibiting high levels of alexithymia were more prone to engage in self-harming behaviours, suggesting that NSSI might serve as an externalising coping mechanism for the overwhelming and poorly understood internal emotional states that these individuals experience.^10^ The inability to effectively process and communicate emotions can, in certain scenarios, culminate in behaviours that offer immediate yet transient relief, such as NSSI.

Peer victimisation, encompassing experiences such as bullying, teasing and social exclusion, remains a pervasive issue in adolescent populations and has garnered significant research attention owing to its detrimental effects on mental well-being.^11^ Adolescents exposed to consistent peer victimisation often report increased feelings of isolation, depression and anxiety.^12^ In the context of NSSI, peer victimisation not only emerges as a direct risk factor but may also play a part in exacerbating or mediating the effects of other risk factors.^13^ Myklestad and Straiton^14^ demonstrated that adolescents who were victims of bullying showed higher rates of self-harm, especially when this risk factor was combined with others such as family maltreatment.

However, an important gap remains in our understanding of how these two factors might interactively influence NSSI behaviours. Although studies^15,16^ have documented the individual contributions of alexithymia and peer victimisation to NSSI, few have delved into their combined effects. Adolescents with high alexithymic traits who also experience peer victimisation might be at elevated risk for NSSI as they navigate the compound challenges of emotional dysregulation and social adversity. In addition, the unique sociocultural milieu of China, characterised by its distinct educational pressures and familial expectations, amplifies the need to study these interactions in the context of Chinese adolescents.

Another research gap exists regarding nuances of the experiences of different age and education subgroups within this demographic. For instance, a study^2^ mapped global prevalence rates of NSSI but stopped short of providing granulated insight into the varied susceptibilities across age or educational attainment brackets. Given the vital developmental transitions and cognitive shifts that adolescents undergo, understanding the differential vulnerabilities of younger versus older adolescents to NSSI could result in critical insights.

In light of the evident gaps in current literature and the pressing need to understand the combined influence of alexithymia and peer victimisation on NSSI within the Chinese adolescent framework, the primary objective of this study was to explore the relationships among alexithymia, peer victimisation and NSSI in a cohort of Chinese adolescents. This research endeavours not only to contextualise the findings within the literature but also to bring forth new insights that could guide preventive strategies and interventions. This study also explores how age and education levels interact with alexithymia and peer victimisation.

## Method

### Participants

Data for the present study were drawn from the Chinese Adolescent Depression Cohort. Participants were sourced from out-patient psychiatric clinics and in-patient wards across 14 hospitals in nine provinces within China, between December 2020 and December 2021. The initial cohort included 2243 adolescents. Eligibility criteria stipulated that participants must be aged between 12 and 18 years, have completed a minimum of 6 years of formal education, fulfil the DSM-5 criteria for NSSI based on clinical assessments, and provide written informed consent alongside parental or guardian consent. Exclusion criteria included a prior personal history of mental illness, a family history of mental illness among first-degree relatives, the presence of an organic disease (e.g. neurological disorders, genetic syndromes), and active psychiatric symptoms such as hallucinations or delusions. After applying these criteria, the final sample under analysis comprised 605 adolescents.

### Procedure

Each participant underwent an evaluation conducted by psychiatric directors; the duration of the assessment depended on the participant's psychological condition, but it typically lasted around 15 min. Subsequent to successful evaluation, participants were guided to complete a questionnaire supervised by graduate students specialising in psychology or psychiatry. Prior to initiating the study, all involved researchers underwent specialised training to ensure uniformity and rigour in the evaluation process. The questionnaires were administered using tablet computers in a quiet ward and took approximately 30 min to complete. Informed consent was obtained from all participants in writing before the study commenced. Ethical approval for the study was granted by the Institutional Review Board of Shenzhen Kangning Hospital under ethics approval no. 2020-k021-02.

The authors assert that all procedures contributing to this work comply with the ethical standards of the relevant national and institutional committees on human experimentation and with the Helsinki Declaration of 1975, as revised in 2008. All procedures involving human subjects/patients were approved by the Institutional Review Board of Wenzhou Seventh People's Hospital (EC-KY-2022048).

### Measures

#### Demographic information

Data on demographics were collected through self-administered questionnaires that queried participants’ age, gender and educational attainment. Age and education were treated as continuous variables, whereas gender was dummy-coded as 0 for males and 1 for females.

#### Self-injury

NSSI was gauged using the Functional Assessment of Self-Mutilation, a self-report measure formulated by Lloyd.^17^ This instrument comprises 22 items probing the frequency and methods of self-mutilation in the preceding 12 months and an additional 11 items exploring the underlying reasons. This scale has good reliability and validity in the Chinese population.^18^ A higher aggregate score denotes more frequent NSSI. In the current study, the Cronbach's alpha coefficient for the Functional Assessment of Self-Mutilation was found to be 0.81.

#### Alexithymia

The Toronto Alexithymia Scale (TAS-20) was used to measure levels of alexithymia among the participants. This scale consists of 20 items evaluated on a five-point Likert scale. The TAS-20 is subdivided into three subscales: difficulty identifying feelings, difficulty describing feelings and externally oriented thinking. Higher scores indicate more pronounced alexithymia.^19^ The Chinese versions of the TAS-20 used in this study have been validated in Chinese populations and shown to have good reliability and validity,^20^ with Cronbach's alpha = 0.78.

#### Peer victimisation

The Peer Victimization Questionnaire (PVQ) was used to assess experiences of peer victimisation. This self-report measure consists of 16 items scored on a five-point Likert scale. Higher scores indicate elevated levels of peer victimisation. The questionnaire demonstrated satisfactory reliability and validity in prior research,^21,22^ with a Cronbach's alpha coefficient of 0.92 in this study.

#### Statistics analysis

Descriptive statistics, including means, standard deviations and percentages, were computed to summarise the demographic attributes of the sample. One-way analysis of variance (ANOVA) tests were used to compare alexithymia groups based on peer victimisation and NSSI scores, followed by *post hoc* Tukey tests for pairwise comparisons. Before the ANOVA, the assumption of homogeneity of variances was tested using Levene's test; the results showed that PVQ scores violated this assumption, whereas the remaining variables met it. Therefore, a square root transformation was applied to PVQ scores, after which Levene's test indicated no significant violation of homogeneity. Bivariate correlations were used to investigate associations among all variables. Subsequently, a multiple linear regression model, deploying the enter method, was used to identify predictors of NSSI. Factors of alexithymia, subscales of peer victimisation, age, gender and education level were incorporated as independent variables. Multicollinearity was scrutinised using variance inflation factors.

Mediation analysis was executed through structural equation modelling using maximum likelihood estimation. Variables such as childhood trauma and peer victimisation were treated as latent variables, indicated by observed variables. Model fit was ascertained using comparative fit index (CFI), normed fit Index (NFI) and root mean square error of approximation (RMSEA). Bootstrapping with 1,000 samples was used to estimate 95% confidence intervals for indirect effects.

All statistical procedures were conducted using SPSS version 26 and R version 4.2.1. All tests were two-tailed, and a *P*-value threshold of less than 0.05 was considered to indicate statistical significance.

## Results

### Descriptive statistics

The present study encompassed 605 participants, with a mean age of 14.95 years (s.d. = 1.60). Females constituted the majority of the sample (*n* = 476, 78.70%), whereas males comprised 21.30% (*n* = 129). Participants averaged 9.01 years of education (s.d. = 1.73). On the alexithymia scale, the *M* score was 67.38 (s.d. = 10.70). Peer victimisation had an *M* score of 9.74 (s.d. = 9.10), and for NSSI, the *M* score was 11.51 (s.d. = 8.67).

One-way ANOVA was conducted to examine differences in age, years of education, peer victimisation and NSSI across non-alexithymia, borderline alexithymia and alexithymia groups, as classified by the TAS-20. As shown in Table 1, significant differences emerged in NSSI scores among the groups (*F*_(2, 602)_ = 8.30, *P* < 0.001, *η²p* = 0.03). Tukey HSD *post hoc* analysis revealed that the alexithymia group had significantly higher NSSI scores compared with the non-alexithymia group (*P* < 0.001). Similarly, significant group variations were observed for peer victimisation (*F*_(2, 602)_ = 9.80, *P* < 0.001, *η²p* = 0.03). Subsequent *post hoc* tests established that the alexithymia group experienced greater peer victimisation compared with both the non-alexithymia and borderline groups (*P* < 0.05). The non-alexithymia group averaged fewer years of education than the borderline group, although no significant differences were detected in age and gender distribution (*P* > 0.05).

**Table 1:** One-way ANOVA between alexithymia groups (mean ± s.d.)

Variable	Non-alexithymia (*n* = 48)	Borderline-alexithymia (*n* = 86)	Alexithymia (*n* = 471)	*F*	*P*	*η^2^p*
Age, years	14.87 ± 1.70	15.28 ± 1.61	14.90 ± 1.58	2.09	0.12	0.01
Edu years	8.62 ± 1.85	9.57 ± 1.65	9.11 ± 1.72	4.89	0.01	0.02
Peer victimisation	2.09 ± 1.71	1.96 ± 1.62	2.79 ± 1.68	11.57	<0.001	0.04
NSSI	7.54 ± 6.94	9.88 ± 9.02	12.22 ± 8.64	8.30	<0.001	0.03

ANOVA, one-way analysis of variance; NSSI, non-suicidal self-injury.

### Correlation analysis

Table 2 displays bivariate correlations among all study variables. NSSI exhibited positive correlations with alexithymia and its subscales (*P* < 0.05), suggesting that elevated NSSI behaviours were associated with increased alexithymia. Similarly, higher NSSI levels were linked to greater experiences of peer victimisation and its subscales (*P* < 0.001). Negative correlations were observed between age and NSSI, alexithymia and peer victimisation (*P* < 0.01). Education level was inversely related to NSSI and peer victimisation experiences (*P* < 0.01).

**Table 2:** Matrix of correlations among alexithymia, peer victimisation, demographic variables and NSSI

	1	2	3	4	5	6	7	8	9	10	11	12
1 NSSI	–											
2 Alexithymia	0.27***	–										
3 DIF	0.30***	0.91***	–									
4 DDF	0.22***	0.85***	0.76***	–								
5 EOT	0.08*	0.57***	0.26***	0.24***	–							
6 Peer victimisation	0.37***	0.21***	0.20***	0.16***	0.10*	–						
7 P1	0.34***	0.14***	0.13***	0.09*	0.10*	0.78***	–					
8 P2	0.31***	0.20***	0.20***	0.19***	0.05	0.84***	0.53***	–				
9 P3	0.27***	0.15***	0.16***	0.10*	0.08*	0.86***	0.55***	0.62***	–			
10 P4	0.33***	0.19***	0.18***	0.16*	0.11**	0.84***	0.58***	0.58***	0.65***	–		
11 Age	−0.18***	−0.10***	−0.05	−0.10*	−0.13**	−0.20***	−0.21***	−0.13***	−0.18***	−0.16***	–	
12 Edu	−0.12***	−0.05	−0.02	−0.04	−0.09*	−0.18***	−0.18***	−0.10***	−0.18***	−0.18***	0.81***	–

NSSI, non-suicidal self-injury; DIF, difficulty identifying feelings; DDF, difficulty describing feelings; EOT, externally oriented thinking; P1, physical victimisation; P2, social manipulation; P3, verbal victimisation; P4, attacks on property; Edu, education level.

* *P* < 0.05, **P < 0.01, ***P < 0.001.

### Multiple regression

To elucidate the predictors of NSSI, a multiple regression analysis incorporating alexithymia, peer victimisation and demographic variables was performed. The model was statistically significant (*F*_(10, 594)_ = 16.27, *P* < 0.001, adjusted *R*^2^ = 0.20). The difficulty in identifying feelings subscale of alexithymia emerged as a significant predictor of NSSI (*P* < 0.001). However, difficulty in describing feelings and externally oriented thinking were not significant predictors (*P* > 0.05). Regarding peer victimisation, significant positive predictors included physical victimisation, social manipulation and attacks on property (*P* < 0.05). Verbal victimisation did not significantly predict NSSI (*P* > 0.05). In demographic terms, age was a significant predictor, with younger participants reporting higher NSSI levels (*P* < 0.05). Neither gender nor education years emerged as a significant predictor (*P* > 0.05). Table 3 contains the complete model results.

**Table 3:** Linear regression model of associations of alexithymia, peer victimisation and demographic variables with NSSI

Model		*β*^a^	s.e.	*t*	*P*	VIF
Independent variable	Alexithymia					
	DIF	0.27	0.06	4.74	<0.001	2.43
	DDF	−0.04	0.06	−0.74	0.46	2.44
	EOT	−0.02	0.04	−0.59	0.56	1.10
	Peer victimisation					
	P1	0.17	0.05	3.58	<0.001	1.75
	P2	0.10	0.05	2.04	0.04	7.92
	P3	−0.04	0.05	−0.65	0.52	2.20
	P4	0.14	0.05	2.55	0.01	2.11
Control variables	Age	−0.16	0.06	−2.53	0.01	3.04
	Gender	−0.01	0.04	−0.20	0.98	1.05
	Edu	0.06	0.06	0.98	0.33	2.94
*F*	16.27***					
Adjusted *R*^2^	0.20					

a. All beta values presented in the manuscript are standardised coefficients.

NSSI, non-suicidal self-injury; DIF, difficulty identifying feelings; DDF, difficulty describing feelings; EOT, externally oriented thinking; P1, physical victimisation; P2, social manipulation; P3, verbal victimisation; P4, attacks on property; Edu, education level; VIF, variance inflation factor.

*** *P* < 0.001.

### Mediation analysis

**Fig. 1:**
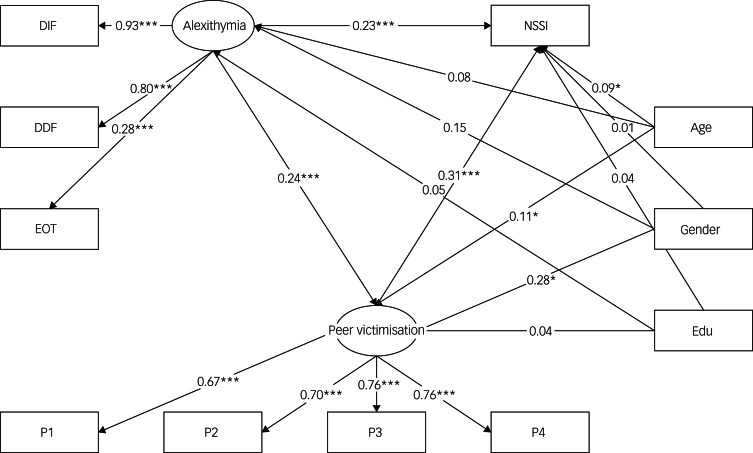
Mediation model path visualisation. DIF, difficulty identifying feelings; DDF, difficulty describing feelings; EOT, externally oriented thinking; P1, physical victimisation; P2, social manipulation; P3, verbal victimisation; P4, attacks on property; Edu, education level; NSSI, non-suicidal self-injury. *P < 0.05; **P < 0.01; ***P < 0.001.

Structural equation modelling was used to assess the mediatory role of peer victimisation in the alexithymia–NSSI relationship, as shown in Fig. 1. Covariates included age, gender and education level. The proposed model exhibited excellent fit to the data (χ²_(33)_ = 71.22, CFI = 0.98, NFI = 0.97, RMSEA = 0.04). In this model, alexithymia was a significant positive predictor of peer victimisation (*β* = 0.24, *p* < 0.001), and peer victimisation significantly predicted NSSI (*β* = 0.31, *P* < 0.001). In addition, alexithymia directly influenced NSSI (*β* = 0.23, *P* < 0.001) even after accounting for the indirect pathway via peer victimisation. Importantly, the mediation effect was statistically significant (*β* = 0.07, 95% CI [0.03, 0.11]), explaining approximately 24.50% of alexithymia's total effect on NSSI. Table 4 elaborates on these effects.

**Table 4:** Effect decomposition for the mediation model

	Effect	95% CI	*Z*	*P*
Direct	0.23	[0.19, 0.27]	5.56	<0.001
Indirect	0.07	[0.03, 0.11]	4.25	<0.001
Total	0.30	[0.22, 0.38]	7.18	<0.001

Direct, direct effect of alexithymia on non-suicidal self-injury (NSSI); indirect, indirect effect through peer victimisation; total, total effect of alexithymia on NSSI.

### Gender invariance of the structural model

To test measurement invariance across genders, a multi-group analysis was conducted comparing an unconstrained model and a model with structural path coefficients constrained to equality. The unconstrained model showed acceptable fit (χ^2^_(56)_ = 101.51, CFI = 0.98, NFI = 0.96, RMSEA = 0.05). The constrained model also demonstrated good fit (*χ*^2^_(70)_ = 106.75, CFI = 0.98, NFI = 0.95, RMSEA = 0.04). The chi-square test revealed that the constrained model did not have significantly worse fitting compared with the unconstrained model (*χ*^2^_(14)_ = 5.24, *P* = 0.98). As constraining the paths to equality did not significantly worsen model fit, this suggests the hypothesised structural model generalised across both genders. Overall, the multi-group analysis provided evidence for measurement and structural invariance of the model across male and female groups.

## Discussion

Our most salient findings indicate that alexithymia significantly predicts both peer victimisation and NSSI. Peer victimisation also emerged as a significant predictor of NSSI and acted as a mediator in the alexithymia–NSSI relationship. Importantly, these relationships were invariant across genders. These findings address a significant gap in the literature by identifying alexithymia and peer victimisation as interconnected risk factors for NSSI in a demographically diverse sample.

### Alexithymia as a multifaceted predictor

Our study reveals a nuanced understanding of how different aspects of alexithymia relate to NSSI. Contrary to the traditional notion that alexithymia operates as a uniform construct,^23,24^ we found that only the ‘difficulty in identifying feelings’ subscale significantly predicts NSSI behaviours. Other components including ‘difficulty in describing feelings’ and ‘externally oriented thinking,’ although recognised as part of the alexithymia construct,^25^ did not exhibit a significant relationship with NSSI.

This selective significance challenges the established, unified model of alexithymia, where all subcomponents are generally presumed to contribute to emotional dysregulation.^26^ It suggests the need for a more targeted, component-focused approach in research and therapeutic settings. The aim would be to address specific emotional deficits rather than treating alexithymia as a monolithic construct. Further, the specific impact of the ‘difficulty in identifying feelings’ subscale also expands our understanding of emotional regulation theories.^27^ These theories propose that identifying and labelling emotions are foundational to regulating them effectively. Our results imply that failing to identify feelings can lead to emotional dysregulation and thereby contribute to maladaptive coping mechanisms such as NSSI.^28,29^ This new insight refines existing theories by pinpointing which aspects of alexithymia are most relevant in the context of NSSI, thus filling a significant gap in the literature.

The lack of significant predictive power in dimensions such as ‘difficulty in describing feelings’ and ‘externally oriented thinking’ may stem from the nature of these components in relation to coping mechanisms. Whereas difficulty in identifying feelings directly affects recognition and understanding of emotional states, which are crucial for emotional regulation,^30^ the other dimensions might influence other areas of functioning that are not directly linked to NSSI. This suggests the need for a more targeted, component-focused approach in research and therapeutic settings.

### Peer victimisation as a mediator

Our findings indicate that alexithymia is not merely a direct contributor to NSSI but also influences it via the path of increased peer victimisation. This suggests that alexithymia's emotional dysregulation has multi-level consequences: it not only directly exacerbates coping mechanisms such as NSSI^31^ but also renders individuals more vulnerable to external stressors such as victimisation.^32^ This structure is deeply rooted in the frameworks of emotion regulation theory^33^ and social interaction theory,^34^ which collectively elucidate the interplay among emotional processing deficits, social adversities and self-harming behaviours. This points to a vicious cycle wherein emotional dysregulation increases vulnerability to external stressors, which in turn exacerbate maladaptive coping mechanisms – a loop that is particularly concerning in the context of adolescent psychological development. Furthermore, the mediatory role of peer victimisation introduces a social dimension to the emotional struggles encapsulated by alexithymia. Alexithymia, often considered an intrapersonal issue, seems to manifest as interpersonal problems through increased susceptibility to peer victimisation. This extends our understanding of alexithymia from being merely a personal emotional limitation to also being a social vulnerability, echoing theories that link emotional intelligence to social competence.^35^

### Demographic factors

Whereas age emerged as a significant predictor, with younger adolescents reporting higher levels of NSSI, gender and years of education did not significantly influence these behaviours. This finding challenges some prevalent notions in the field. The heightened vulnerability of younger adolescents to NSSI underscores the critical nature of early adolescence as a period of heightened emotional and social challenges. The lack of significant gender differences in our study means it diverges from previous research that has often highlighted gender-based disparities in NSSI prevalence and patterns, suggesting a potential cultural nuance in the Chinese adolescent context.^36^ Moreover, the non-significant impact of education years on NSSI implies that educational attainment, contrary to expectations, may not be a protective factor against self-injurious behaviour in this demographic. This insight calls for a more nuanced understanding of the role of education in emotional and mental health, particularly in the context of adolescent development. Overall, these demographic insights contribute a unique perspective to our understanding of NSSI, emphasising the need for age-specific interventions and a re-evaluation of the role of gender and educational influences in shaping self-injurious behaviours.

### Cultural considerations

The findings of this study must be contextualised within the broader Chinese cultural framework, especially considering norms around emotional expression. In Chinese culture, there is often a strong emphasis on maintaining harmony and collective well-being, which can sometimes lead to the suppression of personal emotions for the sake of social stability.^37^ This cultural norm might contribute to higher instances of alexithymia, as individuals might find it challenging to identify and articulate their emotions in a context that values emotional restraint. In addition, the collectivistic orientation of Chinese society, which prioritises group cohesion over individual expression, may further exacerbate difficulties in emotional processing and communication.^38^ This cultural backdrop could explain the heightened levels of alexithymia observed in our sample and its subsequent impact on NSSI behaviours. It is therefore imperative for interventions targeting NSSI in Chinese adolescents to consider these cultural nuances, emphasising the development of emotional literacy in a manner that respects and integrates these cultural values.

### Limitations

Our study was not without limitations. First, the use of self-report measures may introduce biases,^39^ particularly considering the sensitive nature of topics such as NSSI and peer victimisation. Second, the findings, although instructive for understanding the Chinese adolescent population, have limited generalisability to other cultural or age demographics. The unique sociocultural landscape, educational pressures and familial expectations in China may cause certain dynamics to manifest differently than in other regions. Third, the cross-sectional design of our study constrains the extent to which we can make causal inferences, as it captures data at a singular point in time, hindering the establishment of temporal or causal relationships.^40^ Fourth, a notable limitation of our study was the disproportionate gender ratio, with females representing 78.70% of the sample. This skewed distribution could have introduced gender biases into the results. To mitigate such biases, we tested for measurement invariance in our structural equation model and found the relationships to be consistent across genders. This statistical control lends credibility to our findings being generalisable and not an artifact of the imbalanced sample. However, the heavily female-skewed composition warrants caution in interpreting and applying the results. Females may differ from males in terms of socioemotional factors relevant to NSSI, alexithymia and peer victimisation. Last, we realise the distinct characteristics of a clinical sample in our study, which provides detailed insights into NSSI in adolescents seeking medical attention but may not fully represent the broader adolescent population. To enhance representativeness, we sourced our sample from various hospitals across nine provinces in China, covering diverse demographics. However, future research should include a general population sample for a comprehensive understanding of NSSI behaviours across different adolescent subgroups.

### Implications

This study elucidates the complex relationships among alexithymia, peer victimisation and NSSI in Chinese adolescents, with a particular emphasis on the ‘difficulty in identifying feelings’ aspect of alexithymia as a key predictor of NSSI. The role of peer victimisation as a mediator of this relationship underscores the interplay of emotional and social factors in NSSI behaviours. The findings have important implications for mental health interventions and educational strategies. Tailored emotional literacy programmes addressing alexithymic traits, alongside comprehensive anti-bullying initiatives, could be effective in mitigating NSSI risks. Policies should support integrating these elements into educational settings and training for professionals. However, despite the contributions of the present study, its limited generalisability calls for further research in diverse contexts.

## Data Availability

The data that support the findings of this study are available on request from the corresponding author, W.-J.Y.
