# Alexithymia and peer victimisation: interconnected pathways to adolescent non-suicidal self-injury – ERRATUM

**DOI:** 10.1192/bjo.2024.39

**Published:** 2024-03-18

**Authors:** Qian-Nan Ruan, Linhui Liu, Guang-Hui Shen, Yu-Wei Wu, Wen-Jing Yan

**Keywords:** Alexithymia, non-suicidal self-injury (NSSI), peer victimisation, adolescents, emotional dysregulation, erratum

This article was originally published with a note assigning equal contribution to the wrong authors. This has now been corrected and this erratum published.

The publisher apologises for the error.
